# Anti-N-methyl-D-aspartate receptor encephalitis associated with an ovarian teratoma: two cases report and anesthesia considerations

**DOI:** 10.1186/s12871-015-0134-5

**Published:** 2015-10-16

**Authors:** Haiyang Liu, Minyu Jian, Fa Liang, Hongli Yue, Ruquan Han

**Affiliations:** Department of Anesthesiology, Beijing Tiantan Hospital, Capital Medical University, No. 6 Tiantan Xili, Beijing, 100050 China

**Keywords:** Anesthesia, Teratoma, Anti-NMDAR encephalitis

## Abstract

**Background:**

Anti-N-methyl-D-aspartate receptor (NMDAR) encephalitis is an immune-mediated syndrome caused by the production of anti-NMDAR receptor antibodies. The syndrome characterised by psychosis, seizures, sleep disorders, hallucinations and short-term memory loss. Ovarian teratoma is the confirmed tumour associated with anti-NMDAR antibodies. The patients with anti-NMDAR encephalitis complicated by ovarian teratoma require surgical treatment under general anesthesia. NMDARs are important targets of many anesthetic drugs. The perioperative management and complications of anti-NMDAR encephalitis, including hypoventilation, paroxysmal sympathetic hyperactivity (PSH) and epilepsy, are challenging for ansthesiologists.

**Case presentation:**

This report described two female patients who presented for resection of the ovarian teratoma, they had confirmed anti-NMDAR encephalitis accompanied by ovarian teratoma. Two patients received gamma globulin treatments and the resection of the ovarian teratoma under total intravenous anesthesia. They were recovered and discharged on the 20^th^ and 46^th^ postoperative day respectively.

**Conclusions:**

There is insufficient evidence about the perioperative management, monitoring and anesthesia management of anti-NMDAR encephalitis. This report was based on the consideration that controversial anesthetics that likely act on NMDARs should be avoided. Additionally, BIS monitoring should to be prudently applied in anti-NMDAR encephalitis because of abnormal electric encephalography (EEG). Anesthesiologists must be careful with regard to central ventilation dysfunctions and PSH due to anti-NMDAR encephalitis.

## Background

Anti-N-methyl-D-aspartate receptor (NMDAR) encephalitis is an immune-mediated syndrome characterised by psychosis, seizures, sleep disorders, hallucinations and short-term memory loss [1, 2]. This syndrome has been predominantly described in young females (81 %), and ovarian teratoma is the confirmed tumour associated with anti-NMDAR antibodies [3]. One multi-centre prospective epidemiological study demonstrated that anti-NMDAR encephalitis accounted for 4 % of all encephalitis cases [4]. Anti-NMDAR encephalitis has gradually attracted the attention of anesthesiologists because patients with anti-NMDAR encephalitis complicated by ovarian teratoma require surgical treatment under general anesthesia. Here, we describe two female patients who presented for laparoscopic resection of the ovarian teratoma, they had confirmed anti-NMDAR encephalitis accompanied by ovarian teratoma.

## Case presentation

### Case 1

A 31-year-old previously healthy woman (165 cm, 45 kg, ASA I-II) was brought to the emergency room with confusion, agitation, and auditory hallucinations after being admitted to another hospital with seizures and agitation. Intracranial infection was suspected; thus, sedation and appropriate antibiotic treatment were started. The following day, she suffered from hallucinations and developed a regular, involuntary twitch in her right upper limb, mouth, tongue and masticatory muscles.

On admission, viral encephalitis was still suspected, a sedative was given to prevent confusion and agitation, and antiretroviral therapy was started with poor effect. Her computed tomography (CT) and magnetic resonance imaging (MRI) results appeared normal. Cerebrospinal fluid (CSF) and blood analyses showed no significant specific abnormalities. Electroencephalography (EEG) revealed a continuous 1–1.5 Hz sharp and slow mixed wave in the front and central temporal area. On day 7 after admission, other tests (Table 1) revealed anti-NMDAR encephalitis associated with a left ovarian teratoma. Anti-NMDAR encephalitis was diagnosed, and gamma globulin (400 mg · kg^−1^ · d^−1^ for 5 days) treatment was then started; a resection of the left ovarian teratoma under general anaesthesia was scheduled.

**Table 1:** Tests for anti-NMDAR encephalitis associated with ovarian teratoma

	Date	Test	Results
Case 1	Apr. 17, 2013	NMDAR antibodies	Serum: positive (+)
			CSF: Strong positive (++)
	Apr. 18, 2013	Pelvic CT	Left ovarian cystic lesions
	Apr. 22, 2013	Pelvic Ultrasound	Left ovarian cyst
	May 9, 2013	NMDAR antibodies	Serum: Weakly positive
			CSF: positive (+)
	May 14, 2013	Anatomopathology	Ovarian mature cystic teratoma
	May 22, 2013	NMDAR antibodies	Serum: Negative (−)
			CSF: Weakly positive
Case 2	June 16, 2014	NMDAR antibodies	Serum: positive (+)
	June 23, 2014	NMDAR antibodies	Serum: Weakly positive
			CSF: positive (+)
	June 26, 2014	Pelvic Ultrasound	Right ovarian cyst
	June 26, 2014	Pelvic MRI	Right ovarian cystic lesions
	July 1, 2014	Anatomopathology	Ovarian mature cystic teratoma
	July 7, 2014	NMDAR antibodies	Serum: Negative (−)
			CSF: positive (+)

The NMDAR antibody results came from Peking Union Hospital; unfortunately, no precise values for NMDAR antibodies were obtained in the test reports. The second patient rejected further testing for NMDAR antibodies after July 7, 2014

**Fig. 1:**
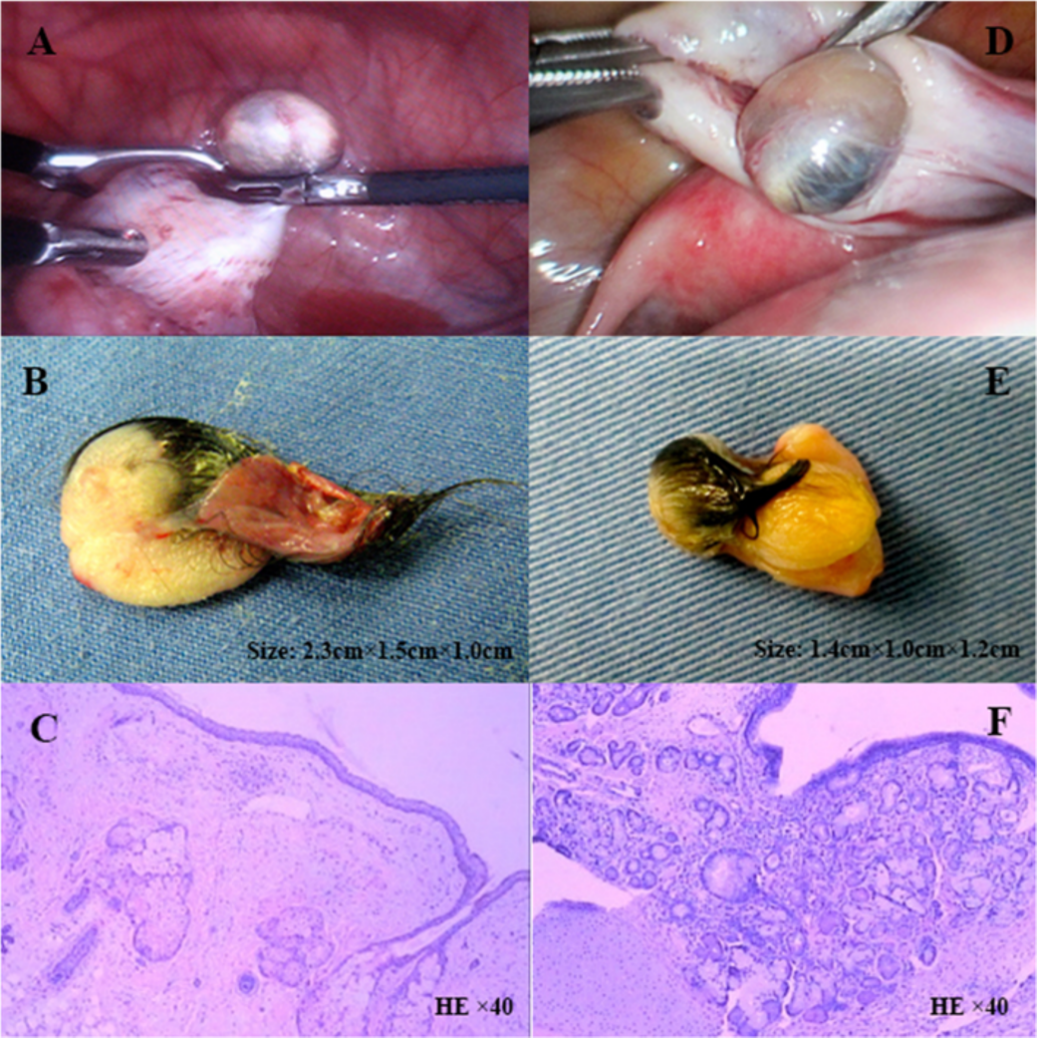
Figures of two cases from laparoscopic resection of ovarian teratoma to pathology. CASE 1: **a** Laparoscopic resection of the left ovarian teratoma; **b** the left ovarian teratoma; **c** pathology of the left ovarian teratoma. CASE 2: **d** Laparoscopic resection of the right ovarian teratoma; **e** the right ovarian teratoma; **f** pathology of the right ovarian teratoma

The resection of the left ovarian teratoma was performed under general anaesthesia on May 2, 2013. Pre-anaesthetic medication was not administered. On arrival to the operating room, the patient’s blood pressure was 105/60 mmHg, her heart rate was 75 beats · min^−1^ and her pulse oxygen saturation was 99 % breathing room air. General anaesthesia was induced with intravenous sufentanil (15 μg), propofol (80 mg) and rocuronium (30 mg) and was maintained with propofol (6 mg · kg^−1^ · h^−1^) and remifentanil (0.1 μg · kg^−1^ · min^−1^). Muscle relaxation was maintained with intermittent rocuronium. The patient’s electrocardiography (ECG), non-invasive blood pressure, and pulse oximetry were monitored. The patient’s intra-operative systolic blood pressure was 100–120 mmHg, and her heart rate was 60–80 beats · min^−1^. The case proceeded uneventfully. Muscle relaxations were reversed, and the trachea was then extubated when the tidal volume (TV) was 400 ml and the patient was able to open her eyes in response to a moderate stimulus. Her physical examination remained unchanged from that performed pre-operatively, and no postoperative complications were evident. The patient was transferred to the neuro-intensive care unit. The durations of surgery and anaesthesia were 1 h: 05 min and 1 h: 45 min, respectively. The total intraoperative blood loss was only 20 ml, the urine output was 300 ml, and the total infusion volume was 1000 ml. The tumour was solid, included hair and cartilage, and was pathologically diagnosed as a mature teratoma (Fig. 1a, b, c).

The patient’s condition gradually improved after surgery. She received another gamma globulin (400 mg · kg^−1^ · d^−1^ for 5 days) treatment, and the course was uneventful. The anti-NMDAR antibody levels in the blood and CSF gradually decreased (Table 1), and the patient was discharged on the 20th postoperative day.

### Case 2

A 22-year-old woman (166 cm, 60 kg) was admitted to another hospital with severe fever (39.0 °C) and headache on May 18, 2014. An upper respiratory tract infection was initially suspected, and an appropriate antibiotic treatment was given for 7 days. Her fever was relieved after aspirin but her recurrent fever and headache did not improve. In the following days, she developed confusion, seizures and a stiff neck. Consequently, she was transferred to our hospital on June 5, 2014 for further evaluation.

**Fig. 2:**
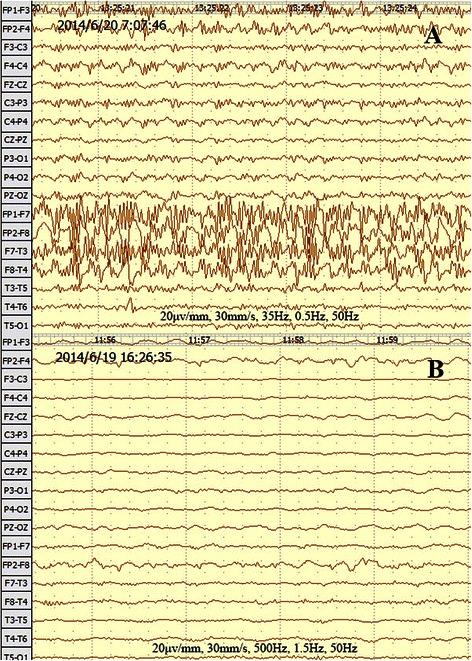
Typical EEG of case 2 before resection of right ovarian teratoma. **a** Persistent high amplitude spikes (3.0–5.0 Hz) in all leads. **b** Generalised rhythmic delta activity

On admission, viral encephalitis was suspected. Appropriate antibiotics and sedation were given to treat encephalitis and seizures, respectively. During the treatment, nasal intubation was performed for excessive oral secretions and pneumonia, and β-receptor blockers were infused to alter sympathetic activity. Her cranial CT and MRI results appeared normal, and the CSF analysis was normal. A chest X-ray confirmed pneumonia, and blood analysis demonstrated leucocytosis associated with pneumonia. A targeted antibiotic was then used against *Staphylococcus aureus* (sputum culture confirmed). A severely abnormal EEG displayed low amplitude waves with slow rhythm in right occipital and posterior temporal lobe and spike waves throughout the brain (Fig. 2a). On day 11 after admission, other tests (Table 1) revealed anti-NMDAR encephalitis associated with right ovarian teratoma. Anti-NMDAR encephalitis was diagnosed, and gamma globulin (400 mg · kg^−1^ · d^−1^ for 5 days) and methylprednisolone (40 mg Q12h) treatment was then started. A resection of the right ovarian teratoma under general anaesthesia was scheduled.

On June 26, 2014, the resection of the right ovarian teratoma was performed under general anaesthesia. Pre-anaesthetic medication was not administered. The patient was transferred to the operating room with a nasal endotracheal tube. Her blood pressure was 123/88 mmHg, her heart rate was 127 beats · min^−1^, and her body temperature was 38.8 °C. Multi-parameter physiological monitoring showed an end-tidal carbon dioxide (etCO_2_) value of 24 mmHg and a respiratory rate (RR) of 32 beats · min^−1^, and arterial blood gas analysis showed a pH of 7.55 and a PCO_2_ of 30 mmHg before induction. Esmolol was infused for sympathetic hyperactivity. Mechanical ventilation (RR = 12, TV = 550) started after induction with sufentanil (15 μg), propofol (80 mg) and rocuronium (35 mg). General anaesthesia was maintained with propofol and remifentanil (0.1 μg · kg^−1^ · min^−1^); the propofol infusion rate was adjusted based on the bispectral index (BIS) (40–50). The patient’s ECG, non-invasive blood pressure, pulse oximetry, body temperature and BIS were monitored. The patient’s intra-operative systolic blood pressure was 90–130 mmHg, her heart rate was 90–120 beats · min^−1^, her body temperature was 38.5–38.8 °C and her BIS was 40–50. The case proceeded uneventfully. Muscle relaxation was reversed, and when the patient’s tidal volume was greater than 400 ml and her pulse oxygen saturation was more than 95 % with room air, she was transferred to the neuro-intensive care unit with a nasal endotracheal tube. The surgery and anaesthesia durations were 35 min and 75 min, respectively. The intraoperative total blood loss was only 10 ml, the urine output was 100 ml, and the total infusion volume was 1000 ml. The tumour was solid, included hair and cartilage, and was pathologically diagnosed as a mature teratoma (Fig. 1d, e, f).

The patient was able to open her eyes and move her upper limbs on command 1 day after surgery. She received another gamma globulin (400 mg · kg^−1^ · d^−1^ for 5 days) treatment after surgery and showed further improvement. She recovered from pneumonia 26 days after admission, and the nasal endotracheal tube was extubated after her ability to swallow recovered. The patient was discharged on the 46^th^ postoperative day.

## Discussion

The well-characterised NMDAR channel requires two NR1 and two NR2 subunits to form a tetramer and is located in the hippocampus, cerebral cortex, basal ganglia and thalamus. NMDAR antibodies in encephalitis show selectivity for NR1 subunits. Ectopic NMDAR expression damages immune tolerance, eventually leading to anti-NMDAR encephalitis. Hughes et al. [5] demonstrated that in patients, NMDAR antibodies cause a selective and reversible decrease in NMDAR surface density and synaptic localisation that correlates with patients’ antibody titres. Anti-NMDAR encephalitis is often accompanied by tumours, particularly teratomas because teratomas contain both nervous tissue and the NMDA receptor subunit, which acts as an antigen to induce antibody expression. The antibodies in the serum and CSF combine with the NMDAR in the basal forebrain, basal ganglia and cervical spinal cord and cause the syndrome, which is characterised by psychiatric disorders, short-term memory loss, dyskinesias and autonomic instability [6].

Body temperature more than 38.3 °C occurs with an incidence of up to 70 % in neuronal damage patients [7]. Many cases of fever of unknown origin have traditionally been classified as central fever. Several studies suggest that one of the key impacts of fever is an increase in neuronal excitotoxicity [8], which also occurs in anti-NMDAR encephalitis. NMDAR antibodies can block NMDAR in the glutamatergic postsynaptic space and in inhibitory GABAergic neurons. The experimental literature and clinical observations both confirmed the negative impact of fever in neuronal damage patients [9]. Antipyretic agents, including acetaminophen, aspirin, and other nonsteroidal anti-inflammatory drugs, are believed to lower the hypothalamic set point and activate the body’s two principle mechanisms for heat dissipation: vasodilation and sweating [10]. We can also use some nonpharmacologic interventions such as external cooling and intravascular cooling to release the fever.

Anesthetics work by interacting with the ion channel targets that regulate synaptic transmission and membrane potentials in key brain and spinal cord regions. The ion channel targets are differentially sensitive to various anesthetic agents [11]. Anesthetics modify the activation of the central nervous system by inhibiting excitability neurons or exciting inhibitory neurons. Importantly, NMDARs mediate excitatory neurotransmission in the nervous system [12]. Various electrophysiological studies have suggested that ketamine and nitrous oxide minimally affect GABA_A_ receptors but inhibit NMDARs [13–15], so they should be avoided to such patients. Propofol was utilised to induce and maintain anesthesia for the two patients in this report. The effect mechanism of halogenated inhaled anesthetics and propofol is not very clear still, no definitive conclusion has been drawn regarding the contribution of NMDAR or GABA_A_ receptors to inhalation anesthetics and propofol. Pryzbylkowski et al. [16] considered halogenated inhaled anesthetics to be appropriate for anti-NMDAR encephalitis patients. Conversely, Hollman et al. [17] reported that the halogenated ethers isoflurane, sevoflurane, and desflurane all inhibited NMDAR to similar degrees at clinically equi-anesthetic concentrations. Further research showed that sevoflurane, isoflurane and xenon inhibited NMDA currents by 12, 31 and 39 %, respectively. It is different that the importance and mechanism of NMDAR in pharmacology of halogenated inhaled anesthetics, such as immobilization, sendation and amnesia [18]. A large number of studies have shown that propofol anesthetises by enhancing GABAergic transmission [19, 20]. Although there are several reports demonstrate that propofol also inhibits NMDAR in clinically relevant concentrations [21, 22], GABA_A_ receptors play a major role in inducing anaesthesia [11]. Propofol was utilised to induce and maintain anaesthesia for the two patients in this report. Ideally, controversial anesthetics that likely act at NMDARs should be avoided.

NMDAR antagonists have been shown to augment analgesia from opioids in acute and chronic settings and to improve opioid-induced hyperalgesia (OIH) caused by opioid exposure [16]. Moreover, the sustained increase in the response of NMDARs via protein kinase-C-mediated manganese removal seem to be the main mechanisms implicated in OIH [23]. However, drawing conclusions regarding whether anti-NMDAR encephalitis patients require more opioids or whether the OIH incidence is decreasing and less NMDARs antagonists are required is difficult, further investigations and researches are expected.

EEG is useful in anti-NMDAR encephalitis, not only for seizure and status epilepticus detection [24] but also for diagnosis [25]. In a study addressing the EEG features of 23 anti-NMDAR encephalitis patients, the main findings were diffuse background slowing with delta slow waves and generalised extreme delta brush [26]. Generalised rhythmic delta activity in anti-NMDAR encephalitis may represent the effect of the antibodies against the NMDAR, leading to reduced NMDA function [27]. Generalised delta activity in case 2 was presented before resection of right ovarian teratoma (Fig. 2b).

A report on anti-NMDAR encephalitis proposed using the BIS to monitor such patients [28]. However, as described above, many anti-NMDAR encephalitis patients show abnormal EEGs, and the value of BIS monitoring in these patients is controversial. The recommended propofol infusion rate is 4.5–9 mg/kg/h combined with opioids [29]. However, a faster rate of more than 10 mg/kg/h was used to maintain the optimal BIS value (40–50) in the second case. An inherent pharmacokinetic and pharmacodynamic variability of propofol certainly exists among patients. Additionally, the therapeutic propofol concentration is highly dependent on the surgical stimulus. Nonetheless, the sensibility of using the BIS value was suspect in the second case because of an abnormal preoperative EEG.

Paroxysmal sympathetic hyperactivity (PSH) is a syndrome of paroxysmal, episodic sympathetic hyperactivity after acquired brain injury that has been recognised for almost 60 years [30]. PSH accompanying anti-NMDAR encephalitis may be associated with glutamate pathway hyperactivity [31]. The patient in the second case presented some clinical manifestations of PSH, including hypertonia and increased HR, RR, BP, temperature and sweating. The first line medications for PSH include most opioids, gabapentin, benzodiazepines, centrally acting α-agonists, and β-antagonists [31]. Esmolol was given to relieve PSH in the second case. Additionally, antihypertensives and anticholinergics should also be used.

Central hypoventilation is a significant clinical feature of anti-NMDAR encephalitis. However, similarly to a report from Vural et al. [32], the patient in the second case suffered from hyperventilation, which was possibly central neurogenic hyperventilation. The inactivation of GABAergic neurons due to an antibody-mediated decrease in NMDA receptors may be involved in the complexity of some abnormal movements via disinhibition of a brainstem central pattern generator [33], which is the possible underline mechanism of central neurogenic hyperventilation. Central ventilatory disorders should arouse the attention of anesthesiologists.

## Conclusions

Currently, there is insufficient evidence about the perioperative management, monitoring and anesthesia management of anti-NMDAR encephalitis. This report was based on the consideration that controversial anesthetics that likely act on NMDARs should be avoided. Additionally, BIS monitoring should to be prudently applied in anti-NMDAR encephalitis. Anesthesiologists must be careful with regard to central ventilatory disorders and PSH due to anti-NMDAR encephalitis.

## Consent

Written informed consent was obtained from the patients for publication of this Case report and any accompanying images. A copy of the written consents is available for review by the Editor of this journal.

## Abbreviations

NMDAR: N-methyl-D-aspartate receptor
PSH: Paroxysmal sympathetic hyperactivity
EEG: Encephalography
ECG: Electrocardiography
etCO_2_: End-tidal carbon dioxide
RR: Respiratory rate
BIS: Bispectral index
TV: Tidal volume
CSF: Cerebrospinal fluid
OIH: Opioid-induced hyperalgesia
